# Temple syndrome diagnosed in an adult patient with clinical autism spectrum disorder

**DOI:** 10.1002/ccr3.1895

**Published:** 2018-11-08

**Authors:** Takuro Kimura, Masayo Kagami, Keiko Matsubara, Shuichi Yatsuga, Rio Mukasa, Chiho Yatsuga, Takako Matsumoto, Yasutoshi Koga

**Affiliations:** ^1^ Department of Pediatrics and Child Health Kurume University School of Medicine Kurume, Fukuoka Japan; ^2^ Department of Molecular Endocrinology, Research Institute National Center for Child Health and Development Tokyo Japan; ^3^ National Hospital Organization Hizen Psychiatric Center Saga Japan

**Keywords:** autistic spectrum disorders, imprinting disease, natural history, Temple syndrome (TS14)

## Abstract

Temple syndrome (TS14) leads to growth failure, precocious puberty, and diabetes mellitus. However, the long‐term prognosis, including the development of social behavior in TS14 patients, remains unclarified. We report the clinical course of a male patient with autism spectrum disorder that received a diagnosis of TS14 at 33 years of age.

## 1 INTRODUCTION

Temple syndrome (TS14), caused by abnormal gene expression of the imprinted genes at 14q32.2, is associated with nonspecific symptoms such as prenatal and postnatal growth failure, feeding difficulty, hypotonia, precocious puberty, obesity, and diabetes mellitus (DM).^1^, ^2^ Genetic causes of TS14 are paternal deletions and an epimutation affecting the 14q32.2 imprinted region, and maternal uniparental disomy of chromosome 14, which lead to hypomethylation of the intergenic differentially methylated region (IG‐DMR) and/or *MEG3*‐DMR.^1^ Nonspecific TS14 clinical features such as prenatal and postnatal growth failure, feeding difficulty, and hypotonia overlap with those of Prader‐Willi syndrome (PWS) and Silver‐Russell syndrome.^1^ Furthermore, some TS14 patients may not receive a diagnosis of TS14 due to nonspecific clinical features and almost normal intellectual development. Because all previously reported TS14 cases were detected in childhood or ascertained by familial studies of children with Kagami‐Ogata syndrome, which is an imprinting disorder as a mirror image of TS14,^1^, ^2^ long‐term prognosis of TS14 including the development of social behavior, language and learning ability, and communication skills remains to be clarified. Here, we report the clinical course of a male patient with clinical autism spectrum disorder (ASD) that received a diagnosis of TS14.

This case study was approved by the Institutional Review Board Committee of Kurume University. Informed consent was obtained from the patient and the patient's parents.

## 2 CASE HISTORY

The male patient was born at 39 weeks, to nonconsanguineous parents. At birth, he was 47.5 cm tall (−0.7 SD) and weighed 2690 g (−1.2 SD) with head circumference of 32.2 cm (±0 SD). He received a clinical diagnosis of PWS because of hypotonia during infancy. After infancy, his hypotonia improved, and he had no regular follow‐ups postinfancy. In early childhood, he exhibited moderate obesity and precocious puberty (pubic hair developed at 9 years of age with a growth spurt); however, no medical intervention was initiated. He graduated from high school without special needs and attended vocational school, but after being rejected from numerous job interviews, he was unemployed. At 23 years of age, he developed body weight gain (body mass index [BMI] = 24.5 kg/cm^2^) and DM (HbA1c, 10.6%). He attempted to lose weight by exercise and insulin therapy for one year, and both obesity and DM improved. At 33 years of age, he visited our hospital to undergo genetic diagnosis and to receive social welfare. He was 159 cm tall (−2.0 SD), weighed 49.9 kg (−1.2 SD), with a BMI of 19.7 kg/m^2^, and head circumference of 56.5 cm (−0.9 SD). He presented as nonobese, with small hands and short stature. There was no hyperphagia; however, he exhibited DM (fasting blood glucose, 432 mg/dL and HbA1c, 14.8%) and dyslipidemia (total cholesterol, 250 mg/dL; HDL‐cholesterol, 46.3 mg/dL; triglycerides, 144 mg/dL; and LDL‐cholesterol, 193.6 mg/dL). He continued exercise and insulin therapy for six more months and both conditions further improved.

During the interview, the patient showed both verbal and nonverbal communication difficulties, including lack of eye contact and diminished facial expression. He talked continuously, ignoring cues from others, and had difficulty reading between the lines. His behavioral inflexibility also restricted his everyday life. To assess his intellectual level and features of ASD, we administered the Wechsler Adult Intelligence Scale (WAIS)‐III to the patient at 33 years of age. His full IQ (FIQ) was 97, verbal IQ (VIQ) was 104, and performance IQ (PIQ) was 88. There was a significant difference between VIQ and PIQ. We also performed the following ASD assessments: the Pervasive Developmental Disorders ASD Rating Scale‐Text Revision (PARS‐TR) for the patient's mother and Autism Spectrum Quotient (AQ) for the patient himself. The PARS‐TR scores were 1 (≥9; ASD‐likely) for early childhood and 4 (≥20; ASD‐likely) for adolescence/adulthood. The total AQ score was 23 (≥26; ASD‐likely). Although both assessment scales scored below the cutoff value, we clinically diagnosed ASD in this patient. Assessment using other tools such as the Autism Diagnostic Observation Schedule (ADOS) and Autism Diagnostic Interview Revised (ADI‐R) is considered to be the gold standard for ASD diagnosis; however, both tests have only recently been adopted within Japan, and testers for both tests are limited. Moreover, the patient was unmotivated for further tests, more so because the tests take nearly 2 hours to complete. Due to this, we did not conduct the ADOS and ADI‐R

**Figure 1:**
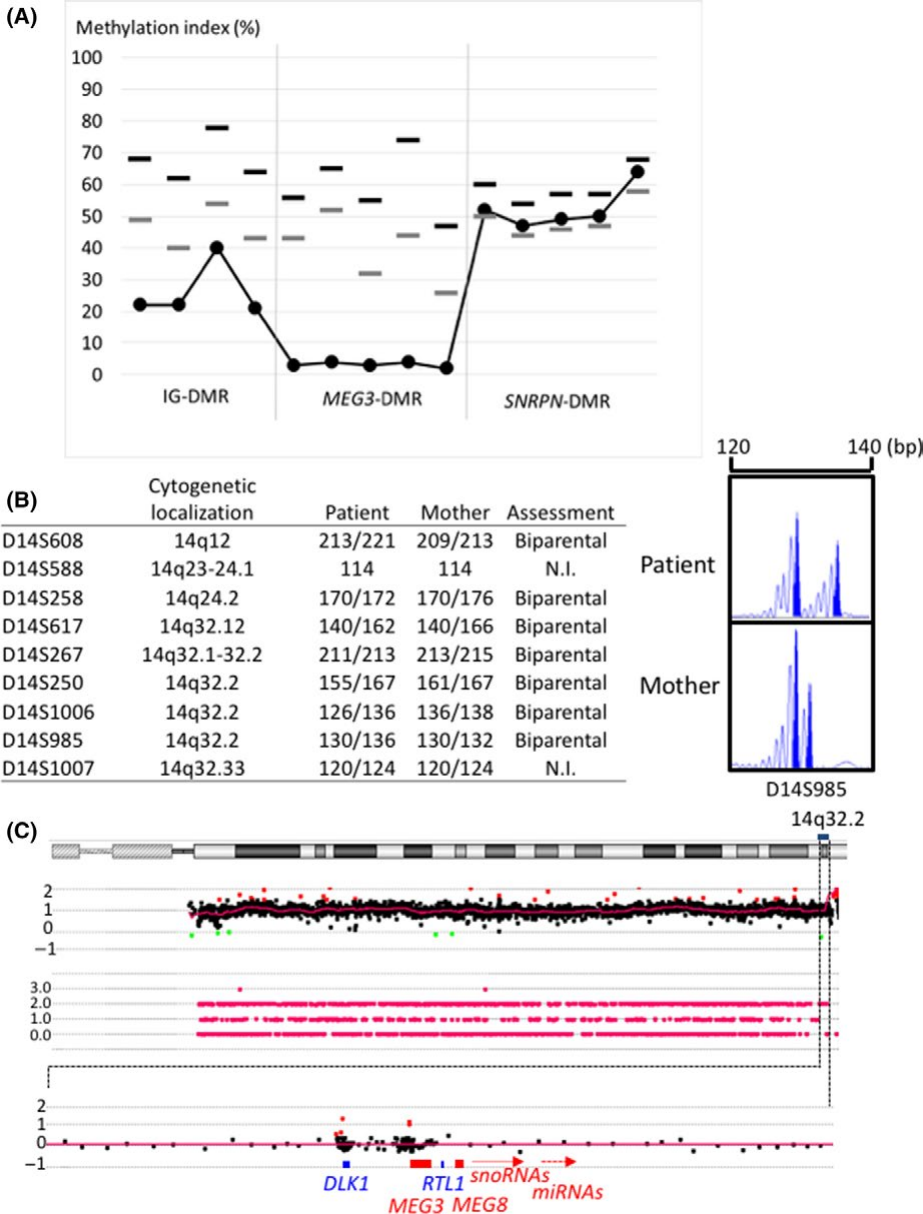
Results of molecular analyses. A, The results of methylation analysis using pyrosequencing: Black and gray dotted lines depict upper and lower limit values of the reference range, respectively. The methylation levels of the IG‐DMR and *MEG3*‐DMR were below the lower limit values of the reference range. B, microsatellite analysis for chromosome 14. Left: summary of microsatellite results. Right: representative microsatellite results. C, CGH +single nucleotide polymorphism (SNP) microarray profiles for chromosome 14. We performed a single nucleotide polymorphism (SNP) array analysis using the SurePrint G3 ISCA CGH +SNP Microarray Kit (catalog number G4890A, Agilent Technologies). NI, not informative

He had normal karyotype, and his fluorescence in situ hybridization analysis with probe including the promoter region of *SNRPN* at 15q11‐13 showed no deletions. For genetic diagnosis, we performed molecular analysis using genomic DNA (gDNA) from leukocytes of this patient and his mother. We first performed methylation analysis for the *SNRPN‐*DMR being the responsible DMR of PWS and the IG‐DMR and *MEG3*‐DMR being the responsible DMRs of TS14 using pyrosequencing as previously reported.^3^ He had hypomethylation of the IG‐DMR and *MEG3*‐DMR and normal methylation levels of the *SNRPN*‐DMR (Figure 1). Subsequently, to determine the genetic cause of hypomethylation of the IG‐DMR and *MEG3*‐DMR, we carried out microsatellite analysis to examine whether he had uniparental disomy (UPD) or not, using gDNA of the patient and his mother. Primer sets for microsatellite analysis were previously reported.^4^ The parental origin of chromosome 14 was biparental (Figure 1). Subsequently, to detect microdeletion or duplication, we performed single nucleotide polymorphism (SNP) array analysis using the SurePrint G3 ISCA CGH+SNP Microarray Kit (catalog number G4890A, Agilent Technologies). The procedure was as described in the manufacturer's instructions. He had no structural abnormalities on chromosome 14. Consequently, we diagnosed TS14 in the patient caused by an epimutation (Figure 1).

## 3 DISCUSSION

This patient was suspected as having PWS due to hypotonia during infancy, but he had no regular follow‐ups postinfancy due to the improvement in his muscle strength. He exhibited postnatal growth failure, precocious puberty, obesity, DM, and ASD, and received a genetic diagnosis of TS14 at 33 years of age. Although some clinical features such as hypotonia, feeding difficulty, and growth failure during infancy overlap between PWS and TS14, intellectual development differs between PWS and TS14 after early childhood. Our patient's FIQ was 97, similar to 90 as the average IQ (DQ) of 11 Japanese TS14 patients caused by UPD(14)mat.^1^ In contrast, the IQ of PWS patients is generally around 65.^5^ In other words, patients suspected with PWS in infancy without intellectual developmental delay but with postnatal growth failure and/or precocious puberty should be clinically suspected with TS14.

In patients with TS14, ASD is a rare comorbidity. An adolescent patient with TS14 comorbid with ASD has been reported; however, there was no detail regarding assessment tools, and the author concluded that the comorbidity was by chance.^6^ Conversely, our patient had difficulties in verbal and nonverbal communication that caused significant impairment in his social functioning and showed inflexibility in daily living. Although he scored below the cutoff point on both PARS‐TR and AQ, his clinical features fulfilled the Diagnostic and Statistical Manual of Mental Disorders (DSM‐5) diagnostic criteria for ASD for all items.^7^ We surmise that his low scores for PARS‐TR and AQ are attributable to the lack of awareness on the part of both the patient and his mother. Assessment using other tools such as the ADOS and ADI‐R in TS14 patients may clarify the characteristics of social behavior of TS14 patients. TS14 patients have no expression of the *DLK1* and *RTL1* genes and excessive maternally expressed genes such as *MEG3*,* MEG8*,* snoRNAs*, and *miRNAs* at the 14q32.2 imprinted region.^1^ Although *DLK1*,* RTL1, MEG3,* and *MEG8s* are expressed in the brain,^8^ the function of these genes in the brain has not been reported. Further accumulation of clinical information related to social behavior, language and learning ability, and communication skills could clarify whether ASD developed as a comorbidity by chance or are clinical features of TS14.

Currently, there is no causal treatment of TS14; however, TS14‐related complication can be treated or prevented. Precocious puberty is a frequent complication and can potentially reduce adult stature. This condition is treatable using a gonadotropin‐releasing hormone (GnRH) analog. Patients with TS14 also may develop juvenile‐onset DM without autoimmune antibodies. DM is typically treated by regulating diet, exercise, and insulin.^9^ Our patient was treated with insulin and exercise, and DM was found to improve thereafter. Other complications, including obesity and hypercholesterolemia, can be treated and prevented.^10^ If our case definitively received a diagnosis of TS14 earlier, obesity, DM, and hypercholesterolemia may have been prevented. Definite diagnosis for TS14 is important for not only the prevention of various complications, but also the identification of problems associated with developmental disorders.

In conclusion, we report a 33‐year‐old male patient who received a diagnosis of TS14, caused by an epimutation‐who had disorders in the development of social behavior. Hypotonic infants with unknown etiology should be considered for genetic analysis of TS14. Additionally, long‐term follow‐up is needed, not only to observe precocious puberty and DM, but also to identify problems associated with developmental disorders such as ASD.

## CONFLICT OF INTEREST

The authors declare that there are no conflict of interests.

## AUTHOR CONTRIBUTION

TK: contributed to the concept and design of the case report and wrote the manuscript. MK: analyzed gene and interpreted gene results, and supervised the case report and reviewed the manuscript. KM: analyzed gene and interpreted gene results. SY: interpreted case report and supervised the case report and reviewed the manuscript. RM: analyzed and interpreted development tests. CY: interpreted development tests. TM and YK: interpreted case report.
